# Chronic Stress and Suicidal Thinking Among Medical Students

**DOI:** 10.3390/ijerph13020212

**Published:** 2016-02-15

**Authors:** Anna Rosiek, Aleksandra Rosiek-Kryszewska, Łukasz Leksowski, Krzysztof Leksowski

**Affiliations:** 1 Public Health Department, Faculty of Health Sciences, Nicolaus Copernicus University in Toruń, Bydgoszcz 85-830, Poland; leksowski@poczta.onet; 2 Department of Inorganic and Analytical Chemistry, Faculty of Pharmacy, Nicolaus Copernicus University in Toruń, Bydgoszcz 85-089, Poland; ola.chemia@wp.pl; 3 Department of Rehabilitation, Faculty of Health Sciences, Nicolaus Copernicus University in Toruń, Bydgoszcz 85-094 Poland; leksowski.lukasz@wp.pl; 4 Department of General Thoracic and Vascular Surgery, Military Clinical Hospital in Bydgoszcz, Bydgoszcz 85-681, Poland

**Keywords:** suicidal thinking, chronic stress, physical activity, mental health problems caused by stress

## Abstract

*Introduction*: The subject of chronic stress and ways of dealing with it are very broad. The aim of this study was to analyze stress and anxiety and their influence on suicidal thinking among medical students. *Materials and Methods*: The study was conducted in the years 2014 to 2015 in Poland, at the Medical University—Nicolaus Copernicus University, Collegium Medicum. The objective of this study was to assess chronic stress and suicidal thinking among students and how students cope with this huge problem. Descriptive statistics and chi-square analyses were conducted to detect differences. *Results*: Analyses showed that students’ life is full of stressors. Students toward the end of their education cope better with stress than students starting their university studies. Chronic stress has a strong impact on mental health and suicidal thinking among students. *Conclusions*: The results of the study confirmed that chronic stress and anxiety have a negative influence on mental health and also confirm a relation to suicidal thinking in medical students. Students cope with stress by listening to music, talking to relatives or people close to them, resting or engaging in sports, with cycling, running and swimming being the most common methods used to affect suicidal thinking.

## 1. Introduction

Scientific research on stress and anxiety offers various perspectives on the issue [1,2]. The increasing pace of life, rushed and competitive lifestyles mean that stress is an integral part of human life. A person in a state of adapting to stress shows behavioral defenses. This leads to changes in one’s cognitive processes and emotional landscape. Stress is a well-known contributor to mood, mental disorders, and suicide risk. Stress is a term often used synonymously with negative life experiences, or life events. The long persistence of an overloading condition, in this case, stress, ultimately leads to mental health disturbances or the appearance of disease [2]. People preparing to engage in the medical profession are affected by especially strong and abundant stress factors. At the outset of education, a medical school student must overcome the stress of the interview. Then there are exams and apprenticeships. To top it all there is contact with lecturers, students and other academic staff. Summing up all these factors, a medical student during the educational process is subjected to constant stress, which she/he must overcome. It is worth noting that not every person (student) is able to cope with this pressure, and that negative life events in this context can confer risk of depression and suicidal thinking.

The diathesis-stress model suggests that people have, to different degrees, predispositions for developing depressive symptoms [3]. In the language of this model, these vulnerabilities are referred to as diatheses. Some people may have more of these diatheses for developing depressive symptoms than other people. However, this model suggests that having a propensity towards developing depressive symptoms alone is not enough to trigger the illness. Instead, an individual’s diathesis must interact with stressful life events (of a social, psychological or biological nature) in order to prompt the onset of the illness. The impact of particular stressors varies across different people. Among medical students, common stressors include: the start of studies, changing of personal habits, place of residence, or eating mode, difficulties in the relationship with their thesis supervisor or partner, and also infections. They can all be powerful enough to cause depressive symptoms in someone with a diathesis for this illness. However, each of these events will impact individuals in a unique manner. A significant loss may be enough to trigger depression in one person, while a very similar loss experienced by another person might not faze them.

“Trend data clearly suggest an increase in levels of stress, depression and anxiety at least since the 1980s. It is worth to consider that one reference found that the average high school student in the year 2000 has the same level of anxiety as the average psychiatric patient did in the 1950s, and those rates have only increased in the last decade [4]”. Also West and Sweeting confirm that increase in levels of stress, depression and anxiety at least since the 1987 in Scotland. Overall, by 1999, a third of adolescent girls in Scotland were reporting symptoms of being depressed as compared to just over a sixth in 1987 [5]. According to the American College Health Association (ACHA) the suicide rate among young adults, ages 15–24, has tripled since the 1950s and suicide is currently the second most common cause of death among college students [6]. That study also found 9.4% of students reported seriously considering attempted suicide at least once in a 12 month period, a marked increase from several decades ago [6].

When we look at lifestyle habits—like eating patterns, sexual activity, sleeping and drinking—we also see evidence of markedly increasing maladaptive patterns. Sleep deprivation in students’ lives or poor sleep patterns are not to be taken lightly and are likely significantly associated with mental health problems. Disordered eating and traumatic sexual behavior has also been well documented to be on the rise of mental health problems among students. In 2006 survey provided by the National Eating Disorders Association (NEDA) found that nearly 20 percent of students had or previously had eating disorders. Also, each year more than 696,000 students between the ages of 18 and 24 are sexually assaulted by another student who has been drinking [7,8]. Also more than 97,000 students between the ages of 18 and 24 are victims of alcohol-related sexual assault or date rape [8]. Depressive symptoms, especially in young people, were connected with traumatic stress and susceptibility to alcohol addiction, drug abuse, crime, and a range of other adverse phenomena [9,10,11,12,13,14,15,16]. Studies indicate that depressive symptoms beginning at an early age could have serious developmental and functional consequences, such as academic failure. It has to be noted, however, that stress can result from positive life events as well.

A large cross-sectional web-based study conducted at the University of Newcastle which investigated the prevalence of common mental health disorders like anxiety (13%), eating disorders (14%), and harmful drinking (8%) found that nearly one third of students reported at least one of these disorders [17]. In another Australian study on 4–17 year olds, 14% of children and adolescents reported depression and mental health problems. Many of these people also suffered from co-morbidities and had a higher risk of suicidal behavior.

According to the Association for University and College Counseling Center Directors 70% of counseling center directors think the incidence of mental health problems on campus is increasing [18]. Rates of anxiety and depressive symptoms have sky-rocketed in the last few decades. A lot of cross-sectional studies suggest that between a quarter and a third of students meet criteria for an anxiety or depressive illness during their college experience [19]. Anxiety is the top presenting concern among students (41.6%), followed by depressive symptoms (36.4%) and relationship problems (35.8%) [18]. Also, on average, 24.5% of counseling centers’ clients take psychotropic drugs [18]. These findings suggest an increased burden of mental health problems on college campuses, suggesting that college students may be facing higher levels of stress than in previous generations.

McCann [20,21,22], the creator of the “fight or flight" model, described how animals and people react to stress or danger. He noted that a sequence of actions occurs in the nerves and glands, preparing the body to defend and fight, or escape to a safe place. According to McCann, the stress response is part of a uniform system of body and mind [20,21]. It is characterized by human reactions to stress as the stimulation of two systems. The first is the pituitary gland that secretes adrenocorticotropic hormones stimulating the adrenal cortex and secretion of corticosteroids—cortisol. This results in increased combustion of fat and protein and reduced inflammation. The second system works by stimulating the sympathetic nervous system, which activates the adrenal medulla secreting epinephrine (adrenaline) and norepinephrine (noradrenaline). These hormones are responsible for the increased activity of the body.

The body’s response after stressful experience, such as a disaster, occurs in five stages. The first phase includes the time of shock, psychological numbness—people do not know what happened. In the second phase—automatic actions—people respond to the disaster, but to a small extent remember their experiences. The next phase gives the victims a sense that they have something that their joint actions have the effect of what is done at the expense of their strength and energy. The fourth phase is fatigue, while everyone is aware of the situation. The last phase takes the longest—people learn to live in the new conditions [23]. Unsuccessful resolution of this process may confer an increased risk for suicidal thoughts or behavior. Fatigue and prolonged stress may heighten risk for suicidal thinking or death by suicide [24].

Situations that threaten health and life are classified as a group of traumatic stressors. These can include: floods, hurricanes, fires, armed conflicts, kidnapping, rape—or broadly defined violence [25]. These are events that are negative and at the same time are impossible to control [19]. Another group of stressors are typical life situations concerning young people, including students. These are presented in Table 1 below.

**Table 1:** The list of life events as stressors.

Range	Life Experience	Average Value of The Stressor
1	Adapting to new activities (like the start of study)	39
2	Outstanding personal achievement	28
3	The beginning or end of academic year	26
4	Changing of housing (leaving home and living in dormitory)	25
5	Changing of personal habits (food, *etc*.)	25
6	Difficulties in the relationship with your supervisor (at work, university teachers)	23
7	The change in the distribution of working hours or the day	20
8	Changing the place of residence (starting life in a new city)	20
9	Changing the hours of falling asleep and waking up	16
10	The change in the number of contacts with family	15
11	Changing eating mode	15

Source: based on [25] with the authors’ own modification. The stressor list was shortened and we extracted only the information pertaining to students.

The presented data show that the value of all stressful life events posed to the young man or woman, at the beginning of his/her career equals 229 points (average value). Presented value critical life stressors, to include experiences of trauma, interpersonal, and occupational/academic events, are cognitive distortions that lead some individuals to be more vulnerable to the influences of negative life events and are important risk factors for suicide. Recent research suggests that events which lead individuals to feel the burdensome responsibilities of professional life may be particularly important.

“Negative occupational and academic events also increase risk for suicidal behavior and suicidal thinking, so it is important to know how students can cope with stress” [26,27]. Previous studies on chronic stress among medical students show that coping plays a central role in adaptation to stressful life events [28]. Coping strategies are the specific efforts, both behavioral and psychological, that individuals employ to master, tolerate, reduce, or minimize stressful events. Coping strategies are classified into active and avoidant coping strategies [29]. “Active coping strategies are either behavioral or psychological responses designed to change the nature of the stressor or how one thinks about it” [29]. Avoidant coping strategies “lead people into activities (such as drugs and alcohol use) or mental states (such as isolating themselves) that keep them from directly addressing stressful events” [29]. Active coping is considered a better way to deal with stress, and students involved in this research also mainly used active coping strategies rather than avoidant strategies. Stressful life events can also contribute to subjective experiences of feeling of stress—an anxious mood state or anxiety disorder.

The authors of this study designed it to collect data on the functioning of students under stress and anxiety in everyday life and the influence of these factors on suicidal thinking among the young. Previous studies of stress and suicidal behavior show that some medical school students experience stress to such a degree that it required clinical attention [30]. Mental health problems, such as depression and anxiety are the main contributors to suicidal thinking among adolescents and young adults. In the face of negative occupational and academic events, stress and suicidal behavior may increase. As such, it is of great importance to determine how students can better cope, and thereby reduce the risk of suicide among young people in Poland. To this end, the authors explored various aspects of stress in a particular group of students (medical students) and their relationship to suicidal thinking.

## 2. Materials and Methods

The aim of this study was to determine the specifics of academic stressors and how students deal with them. Also analyzed are stress and anxiety symptoms among the young and their influence on suicidal thinking. The research was designed to collect data on the functioning of students under stress in everyday life, and also to shows how stress affects the mental health of students of medical schools.

The research problem in this paper was formulated as follows: what is the intensity of the stress experienced by students, what health problems are caused by stress in the research group, how does stress affect mental health and depressive symptoms among young people and how do students cope with stress? The study group were also asked if they treat stress as a disease and whether he/she had thoughts of suicide while being under stress.

It can be assumed that stress felt by respondents is very intense. On average, it amounts to approx. 229 points *per* 1 student. Students cope with stress, and deal with it through means of physical exercise. Our hypotheses are that the cause of the experienced stress are primarily exams, as well as other issues related to studying, university, and change of residence. Another hypothesis is that stress felt by respondents is correlated with suicidal thinking and health problems.

The study was conducted in the largest medical school of Kujavian-Pomeranian province in Poland. The study was conducted during 2014–2015. The study was approved by the ethics committee (No. KB 617/2014). The researchers distributed 300 questionnaires, however, they received only 100 questionnaires back. The sample employed in this study was restricted to medical sciences students from different years, from only one university in Poland, although the sample is likely similar to other university students in Poland, given their similar demographic and clinical characteristics.

The study therefore involved 100 students, including 85 women and 15 men. Most of the students were 21–22 years old. The respondents were represented by faculty: public health—28 people, pharmacy—25 people, medical analytics—24 people, a dietetics—11 people, nursing—12 people. The study selected students from various medical faculties and from different years.

The prevalence of stress in the analysis sample according to demographic data was compared in the study variables, such as gender and year of study. Descriptive statistics (average value, standard deviation, and percentages) were used for summarizing the study and outcome variables. Pearson’s chi-square test for trends was used for observing and quantifying the association between a categorical outcome (*i.e*., the stress level and suicidal thinking) and different study variables. The 95% confidence intervals were calculated and a *p* value of <0.05 was considered significant. The study was designed to show whether people preparing for medical professions know how to deal with stress, and whether stress has negative consequences for their mental health and suicidal thinking already at the start of their education. The responses obtained from questionnaires were analyzed by using descriptive statistics and chi-square analyses to detect differences and relations between the specific variables. The questionnaire (Supplementary 1) was anonymous and consisted of 26 questions relating to the stress of everyday life, the causes of stressful situations, coping with the stress and the effects the stress has on a human body and suicidal thinking. The questions for the study of stress were selected based on the literature, which is given at the end of the article as Supplementary 2. Before filling out the questionnaires, the research group was informed about the study purpose and the reasons why it was important to provide real answers.

### 2.1. Measures

*Perceived stress* was assessed using the 10-item Perceived Stress Scale (PSS) [31], which measures the degree to which a respondent appraises situations in his or her life as stressful. These 10 items use a 5-point Likert scale response format, ranging from “0 = Never” to “4 = Very Often”. Scores for individual respondents were obtained by averaging their responses to all the items of the scale. The internal reliability (Cronbach’s alpha) of the scale in a probability sample of the U.S. population was 0.78 [32].

*Burdens for health* were measured by a scale that was developed for the purpose of this study. Students were asked to what extent they felt burdened by stress and the effects the stress had on them. The internal reliability of the subscales was assessed by Cronbach’s alpha. Table 2 lists the final composition, number of items, and internal reliabilities of the three subscales included in this analysis. Subscale scores were obtained by averaging responses of corresponding items.

**Table 2:** Response Scale and Cronbach’s alpha.

Number of Items	Response Scales	Cronbach‘s Alpha
10	**Perceived Stress:** Cohen‘s perceived stress scale (5 point Likert scale: never–very often)	0.82
4	**Suicidal thinking**: subscale: (5 point Likert scale: always–never)	0.80
4	**The effects of stress on a human body**: subscale (5 point Likert scale: always–never)	0.82
3	**Feeling the burden of stress**: subscale (5 point Likert scale: always–never)	0.79
5	**Burdens for health**: Dichotomous scale (Yes, No)	

The students were also asked how they coped with stress on the medical campus. The most frequent responses were included in the analysis. Correlations between test items as measured by Pearson *r* ranged from 0.35 to 0.66 [33]. Additionally, all subjects completed a demographic questionnaire which comprised questions on their age, gender, place of living (rural/small town or city) and their year of the study and major area of study.

### 2.2. Statistical Analysis

The results were collected using Excel spreadsheets so that evaluations of distributions of assessed variables and analyses of their parameters could be conducted and respondents could be grouped with respect to analyzed characteristics. Subsequently, statistical differences between these groups were assessed using χ^2^. Statistical analysis was carried using Statistics version 10.0.

## 3. Results

### Demograhic Data

The research group consisted of 100 students: 85% were women and 15% men. People from rural areas made up 29% of the respondents; the other respondents were from cities. Most of the students were between 21–22 years old. Information on the fields of study of respondents–public health 28%, pharmacy 25%, medical analysis 24%, 11% dietetics, 12% nursing. The data are based on the full 100 person sample.

Stress for 51% of all respondents is an emotional state, while 42% of respondents claimed that stress is a psychological reaction of the body; the rest of the respondents considered stress as a kind of stimulus. Students of the 3rd year most commonly thought that the stress is a psychological reaction of the body, while the majority of students of the fourth year and first year argued that stress is an emotional state for them (χ^2^ = 10.607; df 4; *p* = 0.031).

Approximately 61% of the respondents claimed that they get upset quickly, 23% of respondents said they are not susceptible to stress, and the rest of the students were not able to give a precise answer.

Chronic stress dominates in students’ lives. Nearly a quarter of students declare that they feel stress almost every day (23% of students), 37% reported wrestling with stress 3–4 times a week, with and an additional 34% of students reporting stress several times a month. The rest of the respondents claimed that they rarely experience stressful situations.

Sixty percent of all respondents reported feeling stress really frequently, which can impact mental health and cause depressive symptoms among the young. Chi-square tests showed that a year of the studies significantly affect the incidence of stressful situations with which the respondents met. The respondents attending the third year, more often struggled with stress than respondents in the last year (χ^2^ = 10.587; df 4; *p* = 0.032).

**Figure 1:**
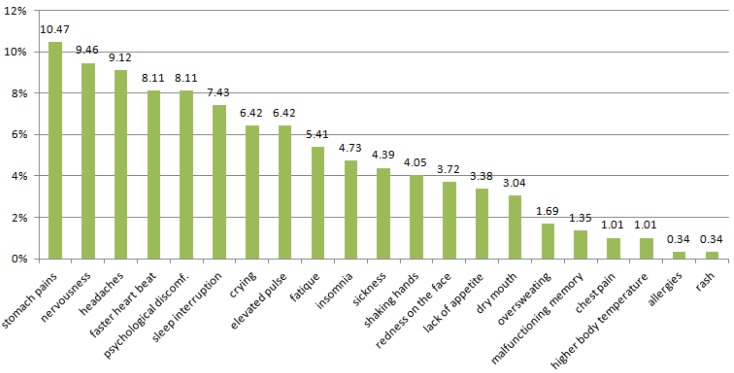
What symptoms of stress do you notice most often in yourself?

**Figure 2:**
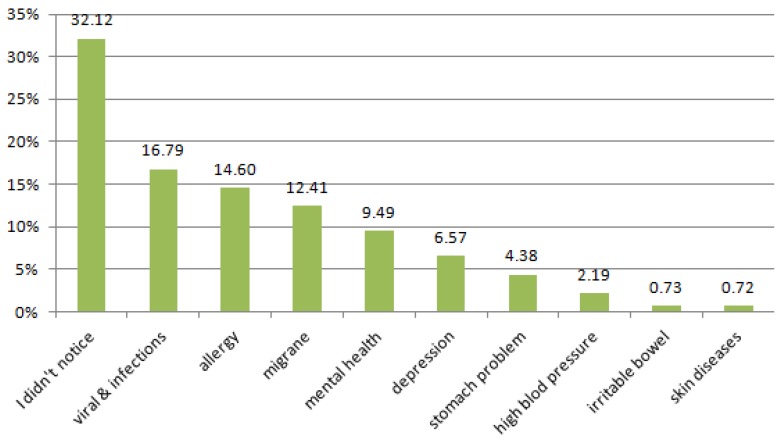
Has stress caused the illness you contracted to be more severe?

Nearly a three quarter of students (72%) declare that they have a circle of friends. Chi-square test showed that friendships may help to reduce suicidal thoughts. Students believe that friendships diminish suicidal thinking. (χ^2^ = 12.057; df 4; *p* = 0.017). However the size of the social circle (friends and colleagues) has not been analyzed in the study. The prevalence of stress in analyzed sample was compared according to demographic data: gender, and year of study, Table 3 below:

The symptoms of stress, noticed by the students include: abdominal pain, nervousness, headache, rapid heartbeat and psychological discomfort, sleep interruption, lack of appetite, fatigue, shaking hands, crying (Figure 1 and Figure 2). Anxiety, depression were revealed in 34.8% of students by symptoms of stress such as psychological discomfort, sleep interruption, lack of appetite, fatigue, shaking hands, and crying.

Some respondents believed that stress exacerbates symptoms of recently-contracted viral and infectious diseases (16.8%) and allergies (14.6%), and also influences mental health (9.49%) and causes depression levels (6.57%; see Figure 2). They declare that many health problems may be affected by chronic stress. However, 80% also feel that to life without stress is an impossibility.

The values of chi-square test—question: do you think that stress is a disease (χ^2^ = 9.265; df 1; *p* = 0.002)/do you think you can live without stress (χ^2^ = 9.265; df 1; *p* = 0.002)—indicate a significant relationship between the described variables. Pearson chi-square test showed two-side statistical significance. It means that chronic stress is a dominant tendency among large group of students in Poland (Table 4).

**Table 3:** The prevalence of stress and demographic data.

Factors		χ^2^	M	SD	*p* level (*p* < 0.05)
**Gender**	Female	6.898	0.610	0.490	0.032
	Male		0.330	0.488	
**Year of study**	1	10.607	1.500	0.707	0.031
	2		2.060	0.747	
	3		1.636	0.809	
	4		2.333	0.795	
	5		2.320	0.802	
**Chronic stress**	1	10.587	2.500	0.707	0.032
	2		1.939	0.747	
	3		1.909	0.701	
	4		2.333	0.795	
	5		2.320	0.802	
**Stress negative influences on live**		12.281	2.910	1.287	0.002
**Social circle**		12.057	2.630	1.812	0.017
**Thoughts of suicide under stress**		7.057	0.660	0.473	0.029
**Stress is a disease**		9.265	0.240	0.427	0.002

**Table 4:** Answers to the question: do you think that stress is a disease/you think you can live without stress.

Question	*n*	Yes	No
Do you think you can live without stress?	*n* = 100	20%	80%
Do you think that stress is a disease?	*n* = 100	24%	76%

Although the students experience health problems caused by stress in their everyday lives, they do not treat stress as a disease. They are aware of the need to combat stress, as this fight can protect them against the development of suicidal thinking and diseases such as: depression and other mental health problems.

Respondents indicated that stress negatively affects their lives and mental health. Approximately 19% of respondents reported that stress contributes to mental health problems (e.g., depressive symptoms). Under the strong influence of severe stress students have suicidal thoughts. The values of chi-square test – question: How often chronic stress negatively influences your life (χ^2^ = 12.281; df 2; *p* = 0.002) and how often do you have thoughts of suicide under chronic stress (χ^2^ = 7.057; df 2; *p* = 0.029)—indicate a significant relationship between the described variables.

**Figure 3:**
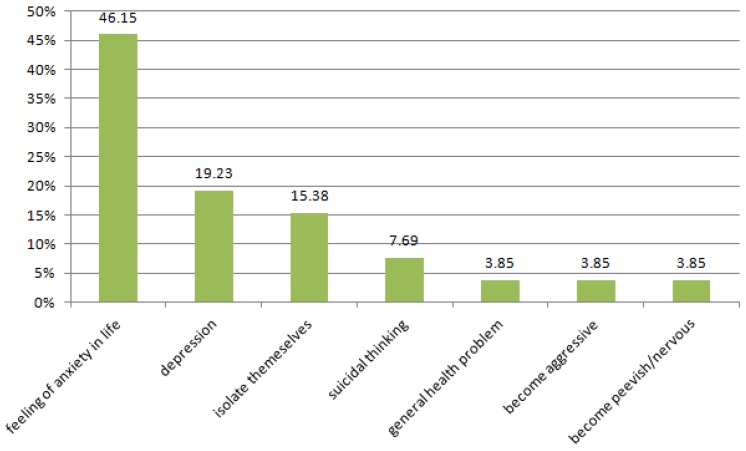
How stress negatively influences your life.

How stress affects negatively the life of students and influences suicidal thoughts—frequency of responses in percent is shown in Figure 3 .

Although stress is not a disease, its presence is associated with suicidal thoughts, depressive symptoms and anxiety among students. As many as 66% of students admit that under stress they have thoughts of suicide. Seventeen percent of respondents declare they rarely have suicidal thoughts under stress and only 17% of students declare that they never thought about suicide.

**Figure 4:**
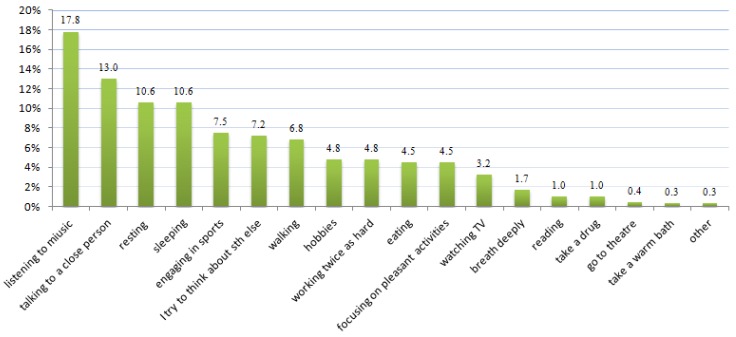
How do you cope with stress?

In order to overcome the stress of everyday duties, students engage in various activities. These include: listening to music, talking with a close person, rest, sleep, sport (cycling, swimming), walking. Physical activities that allow eliminate the effects of chronic stress and suicidal thinking among students are shown in Figure 4 (percentage of students).

## 4. Discussion

Early adulthood is a time typically spent studying at a university. A young man or woman in this period of time is exposed to new life events. This involves feeling severe stress, resulting from undertaking new tasks and challenges. It is also the moment of initiating new commitments. The research shows that during this period of time, students experience stress very strongly. Stress is a common problem among medical students around the globe. Medical students from different parts of the world have been found at risk of psychological stress, mental disorders, and decreased life satisfaction [34]. Chronic stress in this period of life leads them to negative emotional states, depressive symptoms, feeling of anxiety and suicidal thinking.

Caution should however be exercised in generalizing of these findings to all students, although we have no reason to believe that students at this medical university are different from students at other universities in Poland. Depressive symptoms caused by stress were highly prevalent among students in Poland and in 2005 equaled 35% [30]. Also, we have decided to show the data gathered during this research because of the importance of the topic (increasing stress, mental health problems and suicidal thinking ) for public health and the lack of literature concerning stress and mental health issues in the Polish youth population. Our sample is likely similar to other university students in Poland, given their similar demographic and clinical characteristics. Some papers suggested that youth depression symptoms could be triggered by stressful life events such as starting university studies [35,36,37,38,39,40].

Our study confirms that students perceive that stress negatively influences their lives and can lead to serious problems such as depression and suicidal thinking. Many studies indicate that depressive symptoms beginning at an early age can have serious developmental and functional consequences, such as academic failure in future. Also, negative emotional states: depressive symptoms, chronic stress, especially in young people, were connected with susceptibility to alcohol addiction, drug abuse, crime, and a range of other adverse phenomena [9,10,11,12,13,14,15,16].

The collected data suggests that although chronic stress and depressive symptoms among medical students are common [41,42], they are not treated seriously in Poland. Our study shows that feeling of anxiety in students’ lives and depression lead to extremely high rates of suicidal ideation. This situation is alarming for public health as a problem in young society because its mental condition was never before diagnosed and treated in Poland. This state is probably the consequence of the socio-political changes in Poland, the growing pace of life and the pressure of being perfect (the best) at one’s studies. It may lead to a negative impact on mental health. Also, the lack of psychological services for students on medical campuses may lead to high rates of students who are in the state of chronic stress and experience suicidal thoughts. Other researchers also confirm that chronic stress may also lead to the development of anxiety and depressive symptoms [43]. This condition in youths often goes undiagnosed and undertreated. This tendency is also dominant in other countries [44].

The level of perceived stress among students entering education is higher compared to the group of students in their final year of studies. The American Freshman: National Norms Fall 2014 survey found that freshmen arrive at college with less experience in socializing in person and more in time spent online with social media. Students of medical schools are also aware that the ability to cope with stress effectively inhibits the development of many diseases, such as mental health problems (including depression), allergies and other medical problems. This is consistent with other studies conducted in this area [45,46]. The results of the research conducted by Sęk and Pasikowski also confirm this data and hypothesis [47]. Our study also confirms that students in their last year are much better at coping with stress and negative emotional state than their younger colleagues. Students reported that they believed physical activity helps in coping with chronic stress and could be one of the positive ways to reduce stress and suicidal thinking among students.

For students starting school an exam session is a significant stressor along with new life situation that causes fear, reducing effective functioning as well as causing mental health problems and suicidal thinking. Snyder and Wróblewski in their research also confirmed these results [48,49,50]. Exams, public speaking, and personal problems are all stressful experiences. Each student however experiences the stress on an individual basis but each student’s physical or mental health is threatened.

In Poland young people studying medicine are very sensitive; many cannot cope with new problems and most have thoughts of suicide. According to GUS (Main Statistical Office in Poland) data, each year around 1377 young people up to 29 years of age die by suicide. Many of these deaths are related to growing depression and anxiety over everyday life, which is mostly ignored by their schools, environment, and parents. Problems in everyday life also effect physical health. Reoccurring small worries and uncomfortable situations accumulate, and contribute to all sorts of health problems. It is proven that stress contributes to infectious and viral diseases. It also exacerbates the symptoms of chronic diseases such as allergies [20,48,51,52]. Stressful situations are therefore relevant to outbreaks of disease [52]. The speed of occurrence of a disease depends on the degree of adaptability of the stress reaction. Therefore, it is said that the body’s response is adequate to the intensity of the stressor, which is then related to the immunity resources available to a person, that are used in coping with stress [53].

When it comes to the impact of stress on health we should pay particular attention to the immune system and the hypothalamic-pituitary-adrenal axis (HPA axis). It can seriously disrupt the balance of the secretory system, which results in weakening of the immune system. Stimulating the hypothalamus, the anterior pituitary adrenocorticotropin hormone (ACTH) is released and it stimulates the adrenal cortex to secrete cortisol. Cortisol is a stress hormone; it increases blood sugar level and speeds up the metabolism. However, elevated levels of cortisol persisting for a long time become harmful to health and normal functioning of the body [54]. The effect of this is that immunity decreases and susceptibility to various diseases, such as viral infections increases.

The conducted study showed that the effects of stress are very extensive. The prevalence of stress in the study was higher among the female students compared to their male counterparts and showed that females had a higher risk of depressive symptoms than males. This observation is in line with other studies which documented that females’ tendency has been attributed to biological, psychological and even social factors [55,56]. Although some studies have shown that the gender differences did not turn out to be a significant factor in reporting of stress [57,58], other studies confirm results of our research [59,60]. Such variables as being female (*p* < 0.05), year of study (*p* < 0.05), and presence of perceived physical problems (*p* < 0.05), were found as independent significant risk factors for the outcome variables of stress. This result is also confirmed by other researchers [60].

In the case of this research (a group of medical university students) we speak of chronic stress (average value 229 points), and thus all the problems indicate the stress-related diseases (depression, mental health, feeling of anxiety, emotional state and suicidal thinking and also ulcers, infections, allergies).

Chronic exposure to stressful conditions can lead to deterioration of academic performance, loss of memory, poor relationships with peers and family members, and overall dissatisfaction with life [44]. Furthermore, this chronic exposure can also lead to serious health problems such as hypertension, heart attack and stroke, diabetes mellitus, and obesity. Chronic stress also accelerates aging [61], impairs the immune system, suppresses fertility, contributes to digestive problems and loss of appetite, and increases anxiety and depression symptoms that may increase risk for suicidal thinking [62].

Students self-reporting chronic stress also report a number of other mental and physical health symptoms, including psychological discomfort (8.11%), sleep interruption (7.43%), tearfulness (6.42%), fatigue (5.41%), trembling hands (4.05%) and lack of appetite (3.38%). It has been noted that 34.8% of the surveyed students suffered from the above symptoms. The same fact also confirm other researchers [63,64,65].

This study and the experience of other researchers [66] showed that chronic stress contributes to mental health symptoms and suicidal thinking. Up to 66% of medical students admit that they have had thoughts of suicide while under stress, and that endorse the belief that chronic stress negatively affects the lives of students, contributing to anxiety and depressive symptoms and increasing isolation. This huge problem is recognized by The Ministry of Health in Poland, which has prepared a special program to promote mental health at the level of the entire society and prevent of suicides in selected groups at higher risk.

During studies they acquire knowledge of behaviors that help them succeed, hence the stress level and suicide thinking in this group is lower than among students starting school [47]. In addition, students in their last year of studies have developed skills for relieving stress by undertaking various activities.

Conducted research also shows that medical school students chose an active form of fighting stress. Active coping strategies used by students are in line with global tendency [29]. According to students’ self-reporting, sports such as cycling, running and swimming are good ways to cope with stress. This may be because exercise reduces stress and depression and anxiety symptoms. Prior research also suggests that exercise causes the release of “happiness hormones”—serotonin and endorphins—which positively affect the human body and temper emerging health problems. Result of our study confirm other researchers’ data [63,64,65,66]. Ogińska-Bulik [48] also draws attention to the active coping with stress. Physical activity in aspect of coping with stress is the basis for the proper functioning of the body, but the influence of physical activity on eliminating suicidal thinking among young people requires future in-depth research.

The conducted study did not include the association between self-reported size of the social circle (friends and colleagues), and reported way of coping with stress. However, the conducted survey did show that maintaining friendships and bonds with other people is one reported method of overcoming stress in everyday life, and a large group of friends may help to diminish suicidal thinking. Numerous studies conclude that having a large group of friends makes it easier to cope with stress [67,68,69]. It allows one to cope with many stressors such as daily troubles, school and illness, among others. [20,51]. The results of the study so far show that the most frequent users of social support are women, regardless of the cause of stress. The same facts confirm other researchers [54,64,70,71].

This study also suggests that, in the analysis of stress-related diseases, mental health and physical health cannot be considered separately. Unfortunately, however, much of the literature devoted to the subject of stress-related diseases is divided into those affecting mental health and physical health. These two groups of diseases, are however not clinically uniform. In some diseases, stress is a major cause of negative health effects while in others stress is one of many factors. For this reason the list of diseases caused by stress is constantly updated. It’s closely connected to the progress of clinical, biochemical, and patomorphologic research and to the study of the phenomenon of stress and exploration of health problems it brinks. The very respondents did not make such a distinction. Rather, in their descriptions of the methods used to fight stress in order to improve their health (which was not divided into physical and mental health), respondents endorsed physical activity and listening music (which is a form of music therapy widely used to improve mental health).

### Limitations

The sample employed in this study was restricted to medical sciences students from different years, from only one university in Poland. Thus caution should be exercised in generalizing these findings to all students. However, the authors are not aware of any reason to believe that students at this medical university are different from students at other universities in Poland. Depressive symptoms caused by stress were highly prevalent among students in Poland after the political changes. During the years of communism and socialism, the relationship between mental health, work and students’ lives was never researched or analyzed. The presence and frequency of mental health disorders, particularly of depressive symptoms, stress reactions, attempted suicide, even alcoholism and drug abuse, was not publicized. No information was collected on events within or outside the university setting and which could have an effect on chronic stress and suicidal thinking.

This survey was conducted at the university at the end of academic year with exams in the students’ immediate future. It could have influenced the intensity of stress and depressive symptoms felt by students. Although, it is true that suicide risk factors in the general population apply also to medical campuses, here are some unique characteristics in the college group in Poland that require attention and a better understanding. They are intensive and frequent contact students have with ill people (a lot of practical classes), high social expectations, negative consequences for students who decided on an individual studies, frequently changing educational system in Poland and lack of psychological services for overburdened students (having personal difficulties) or problems with studies. Those elements can cause the students from medical universities to be under strong stress. Thus they can have thoughts of suicide more often than students from other countries or other types of universities. Limitations of the study also include these elements as limitations associated with a self-reported, cross-sectional, correlational design and limitations associated with the simplicity of utilized statistics and single-item measurement. Therefore, there is some potential for reporting bias which may have occurred because of the respondents’ interpretation of the questions or desire to report their emotions in a certain way or simply because of inaccuracies of responses.

## 5. Conclusions

Based on a cross-sectional self-reported survey, medical students perceive that exams are the most common source of chronic stress for young people entering adulthood. The level of perceived stress among medical school students is high. However, graduating students cope better with stress than students starting the education, indicating that one can learn to fight stress and the same suicidal thinking decreases. This makes it possible to minimize the negative health effects of chronic stress. The study shows that chronic exposure to stressful conditions may lead to psychological discomfort, mental health problems, depression and anxiety symptoms which might increase risk for suicidal thinking. Additionally, it has been noted that stress contributes to exacerbation of viral and infectious diseases and allergies in the researched group. Students are aware of the need to combat stress and suicidal thinking and of the fact that it can protect them against the development of illnesses such as depression and other mental ailments. In order to reduce suicidal thinking and the symptoms of illnesses caused by stress among students, it turned out that taking up an activity such as sport is very effective. Active coping with stress included listening to music, conversations with loved ones, and sport (cycling, swimming).

The article shows the negative impact of stress on the mental health of students and reveals the problem of mental health among Polish students, which has been downplayed. Thus for students themselves cope with chronic stress, in the absence of professional help from counseling at the university, using active coping strategies. There exists strong association between chronic stress, depressive symptoms and suicidal thinking, and it suggests the need for intervention that will improve stress management at medical and other universities. This topic needs more analysis and research because of its importance for public health and large gaps that exist currently in the literature concerning stress and mental health issues in the Polish youth population.

## Supplementary Materials

The following are available online at http://www.mdpi.com/1660-4601/13/2/212/s1, Supplementary 1: the questionnaire; Supplementary 2: literature.
