# Supplementary material for: Chronic Stress and Suicidal Thinking Among Medical Students
Source: Int J Environ Res Public Health. 2016 Feb 15;13(2):212. doi: 10.3390/ijerph13020212 (PMC4772232; doi:10.3390/ijerph13020212)
Supplement: Supplementary file 1 [file ijerph-13-00212-s001.pdf]

Supplementary Materials: Chronic Stress and Suicidal Thinking Among Medical Students

Anna Rosiek [1,*] Aleksandra Rosiek-Kryszewska [2], Łukasz Leksowski [3] and Krzysztof Leksowski [1,4]

Supplementary 1. Perceived Stress and Burdens for Health

The questions in this scale ask you about your feelings and thoughts during the last month. In each case, you will be asked to indicate by circling *how often* you felt or thought a certain way.

Age ________
Gender (*Circle*): Male Female
Place of living (Circle): rural/ small town/ city
Year of study ____________________________________
Major area of study ____________________________________

0 = "Never" 1 = "Almost Never" 2 = "Sometimes" 3 = "Fairly Often" 4 = "Very Often"

1. In the last month, how often have you been upset because of something that happened unexpectedly?................................. …………..**0 1 2 3 4**

2. In the last month, how often have you felt that you were unable to control the important things in your life? **0 1 2 3 4**

3. In the last month, how often have you felt nervous and "stressed"? **0 1 2 3 4**

4. In the last month, how often have you felt confident about your ability to handle your personal problems? **0 1 2 3 4**

5. In the last month, how often have you felt that things were going your way?.. **0 1 2 3 4**

6. In the last month, how often have you found that you could not cope with all the things that you had to do? **0 1 2 3 4**

7. In the last month, how often have you been able to control irritations in your life?.. **0 1 2 3 4**

8. In the last month, how often have you felt that you were on top of things?............... **0 1 2 3 4**

9. In the last month, how often have you been angered because of things that were outside of your control?.. **0 1 2 3 4**

10. In the last month, how often have you felt difficulties were piling up so high that you could not overcome them? **0 1 2 3 4**

I Burdens for health

	**Yes**	**No**
11. Do you think that stress is a disease?	☐	☐
12. Do you think you can live without stress?	☐	☐
13. Did you have any mental problems before you started studying?	☐	☐
14. Do you think that stress negative affects your health?	☐	☐
15. Do you use some kinds of active substance to cope with stress?	☐	☐

II Feeling the burdened of stress

16.How often do you feel burdened by stress

Always Very often Sometimes Rarely Never

What kind of stressors have you experienced recently (circle only one)?

Failed exam, General problems at the university, Family problems, Health problems, End of relationship, Other____

What is stress for you (circle only one)?

Emotional state, Kind of stimulus, Psychological reaction of the body, Other__________

17. How frequently during your stay at the university are you in stressful situations?

Always Very often, Sometimes, Rarely Never

Detail your answer (circle only one)?

Almost every day	☐	☐	Rarelier than a month
3 - 4 times a week	☐	☐	A few times a month

18. How often do you decide on an active form of eliminating stress?

Always Very often, Sometimes, Rarely Never

III The effects of stress on a human body

19. How often chronic stress negatively influences your life?

Always Very often, Sometimes, Rarely Never

What kind of negative influence do you observe (circle only one)?

I become nervous, I become aggressive, general health problems, feeling of anxiety, I have depression, I isolate myself , I have a suicidal thoughts, other____________________

20. How often symptoms of chronic stress do you notice in yourself?

Always Very often, Sometimes, Rarely Never

What kind of symptoms do you notice most often (circle only one)?

Sleep interruption, Psychological discomfort, Fatigue, Lack of appetite, Elevated pulse, Crying, Headaches, Stomach pain , Faster heart beat, Allergies, Chest pain, Malfunctioning memory, Redness on face, Dry mounth, Shaking hands, Insomnia, Nervousness, Overwseating, Higher body temperature, other____

21. How often chronic stress exacerbated your health problems ?

Always Very often, Sometimes, Rarely Never

To which one (circle only one)?

I didn't notice, Viral infections, Allergy, Mental health, Depression, Stomach problems, Migrane, Diabetes, Hypertension , Skin diseases, Other___________________________

22. How often do you cope with stress by yourself?

Always Very often, Sometimes, Rarely Never

How do you cope (circle only one)?

Watching TV, Eating, Sleeping, Talking to a close person, Listening to music, Working twice as hard, Hobbis, Walking, Trying to think about something else, Resting, Focusing on pleasant activities, Sport, Other___________________________________

If you engage in sport then what kind _____________________________________

IV Suicidal thinking

23. How often do you have thoughts of suicide under chronic stress (Circle only one)?

Always Very often, Sometimes, Rarely Never

24. What kind of stressors evoke negative thoughts and emotions (circle only one)?

Failed exam, General problems at university, Family problems, Health problems, End of relationship, Other____

25. How often do you feel that friendship helps you eliminate suicidal thoughts?

Always Very often, Sometimes, Rarely Never

26. How often do you engage in sports when you have negative thoughts?

Always Very often, Sometimes, Rarely Never

Thank you

Supplementary 2

1 *Your Stress*; Gdańsk Psychological Publisher: Gdańsk, Poland, 2002;
2 Mieczysław, U. *Stress and Antistress—Patomechanism and Health Effects*; MedPharm: Wrocław, Poland, 2009.
3 Trauer, T. *Stress Everything about the Causes, Mechanisms and Consequences of Nervous Tension*; TENTEN: Warsaw, Poland, 1992
4 Zimbardo, P.Z.; Gerring, R.J. *Psychology of Life*; Scientific Publishers PWN: Warsaw, Poland, 2012

© 2016 by the authors; licensee MDPI, Basel, Switzerland. This article is an open access article distributed under the terms and conditions of the Creative Commons by Attribution (CC-BY) license (http://creativecommons.org/licenses/by/4.0/).
